# A Case Series on a Rare Clinical Presentation of Lichen Planopilaris Restricted to the Face

**DOI:** 10.7759/cureus.59987

**Published:** 2024-05-09

**Authors:** Rohit Kothari, Sunmeet Sandhu, Anuj Bhatnagar, Gurpreet K Walia, Therasal Valarmathi

**Affiliations:** 1 Dermatology, Command Hospital Air Force Bangalore, Bangalore, IND; 2 Dermatology, 7 Air Force Hospital, Kanpur, IND; 3 Pathology, Command Hospital Air Force Bangalore, Bangalore, IND

**Keywords:** scarring alopecia, linear, lichen planopilaris of face, hyperpigmented, clinico-histopathological profile, dermoscopy

## Abstract

Lichen planopilaris (LPP) restricted to the face is extremely rare. This case series includes five unique LPP cases that presented with a varied degree of pigmentation and scarring alopecia restricted to the face. We herein describe the clinical characteristics, dermoscopy, and treatment of these histopathologically confirmed facial LPP cases. None of them had lesions anywhere else on the body.

## Introduction

Lichen planopilaris (LPP) is an uncommon follicular variant of lichen planus (LP) classified under primary cicatricial alopecia [1]. It is an autoimmune disease that involves a lymphocytic inflammatory response centered around the follicles, targeting hair follicle antigens. It occurs more commonly in Caucasian and Indian females, involving the vertex and parietal scalp and presenting as patchy/diffuse scarring alopecia. LPP restricted to the face is very rare, with only a few cases reported in the literature. It is described more commonly in males in the fourth to fifth decades. A linear distribution of erythematous or hyperpigmented atrophic macules and/or papules involving the lateral chin and cheek is usually the most common presentation of facial LPP. The exact pathogenesis is not known, and there are no established treatment guidelines for this condition [1-3].

## Case presentation

**Figure 1:**
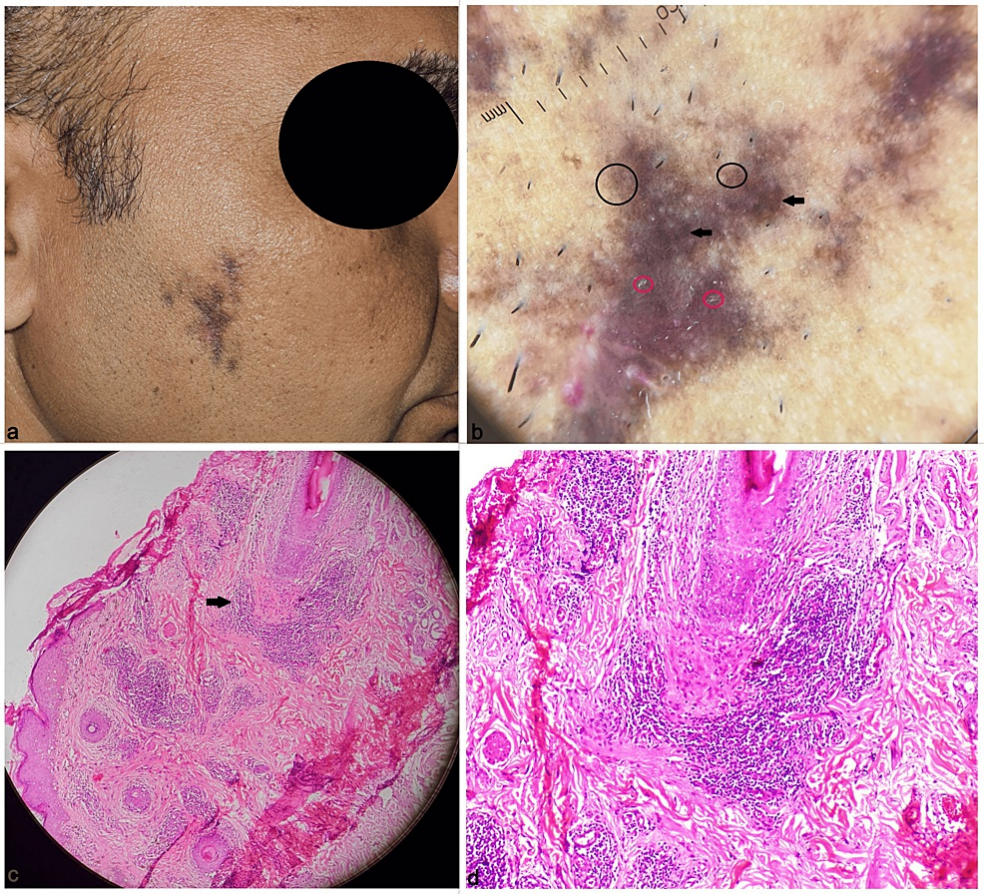
Patient 1: Multiple, irregular, coalescing hyperpigmented to violaceous patches over the right cheek with hair loss and mild atrophy (a); Dermoscopy (Illuco IDS-1100, polarized, 10x) showing brown to violaceous background, significantly reduced hair follicles, pigment pseudo-network, multiple brown dots (black circles), prominent pigment clumps (arrows), and mild perifollicular scaling (red circles) (b); Histopathology showing focal areas of thinning of the epidermis with flattened rete ridges, basal layer vacuolar degeneration with pigment incontinence, perivascular and perifollicular lymphocytic infiltrate (arrow), focal areas of perifollicular fibrosis (H & E, 100x) (c); Focused view showing perifollicular lymphocytic infiltrate (H & E, 400x) (d).

**Figure 2:**
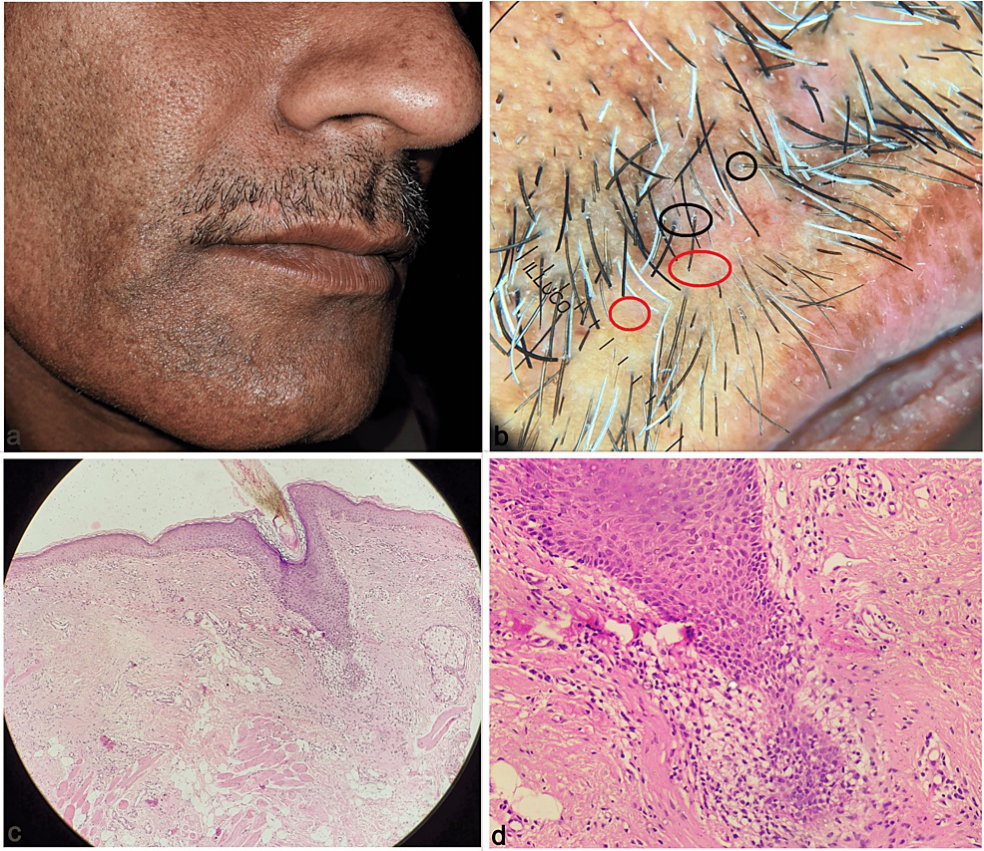
Patient 2: Multiple skin-colored and erythematous atrophic patches of alopecia in a linear distribution over the right side of the mustache (a); Dermoscopy showing foci of brownish white pigmentation with erythema, cicatricial alopecia (red circles), and peripilar casts (black circles) (b); Histopathology showing focal atrophy of epidermis with vacuolar degeneration of the basal layer, perifollicular fibrosis and epithelial atrophy at the level of the infundibulum and isthmus giving rise to an hourglass configuration and underlying dermis showed perivascular inflammatory infiltrate (H & E, 100x) (c); and (H & E, 400x) (d).

All except one were males aged 38-60 years. A 12-year-old girl was the youngest patient in our series as well as among those reported in the literature. All presented with a varied degree of hyperpigmented atrophic macules with scarring alopecia patches restricted to the face (Figures 1a, 2a).

Only two of them had a linear morphology. The most commonly affected site was the chin, which was followed by the upper lips. The cheek and forehead were the least commonly affected. The scalp was normal in all patients. The duration of the lesions ranged from one to six months and none had lesions elsewhere in the body. Although LPP is usually asymptomatic, it may be associated with itching, as noted in two of our patients.

Dermoscopy is an essential adjunctive diagnostic technique in LPP. It was performed in all our patients, and we observed a brown-to-violaceous background, significantly reduced hair follicles, and cicatricial alopecia in all the patients. The majority of them showed mild blotchy erythema, brown dots, and perifollicular scaling. The least common dermoscopy findings were peripilar cast, pigment pseudo-network, pigment clumps, and whitish scar-like areas (Figures 1b, 2b). The dermoscopy of LPP, in general, that commonly involves the scalp also shows similar features except for the more prominent lack of follicular opening, whitish cicatricial areas, perifollicular erythema and scaling, bluish-black pigmentation, and rarely irregular ectatic vessels. Facial LPP may show a pigment pseudo-network that is usually not seen in LPP over the scalp [4].

Histopathology confirmed the diagnosis in all our patients and showed a perifollicular inflammatory infiltrate, basal layer vacuolar degeneration and pigment incontinence, and focal areas of perifollicular fibrosis, corresponding to the erythema and peripilar cast, brown dots and pigment clumps, and scar-like areas, respectively (Figures 1c, 1d, 2c, 2d). Patient 3 had the maximum duration of the disease (six months) and the dermoscopy showed whitish scar-like areas and lacked erythema, signifying advanced disease progressing to fibrosis. Table 1 summarizes the clinical characteristics, dermoscopy, and treatment of all the cases in our case series.

**Table 1:** Clinical characteristics, dermoscopy, and treatment of facial lichen planopilaris patients (+ indicates the presence of the feature mentioned in the corresponding row)

Clinical characteristics	Patient 1	Patient 2	Patient 3	Patient 4	Patient 5	Patient 6
Age (years)	38	47	44	60	12	32
Sex	M	M	M	M	F	M
Site	Cheek	Upper lip	Chin & adjoining neck	Upper lip & chin	Chin & forehead	Cheek
Duration of lesions (months)	Three	One	Six	Four	Three	Four
Presentation	Irregular hyperpigmented to violaceous atrophic patches	Linear skin-colored atrophic patch with mild erythema	Linear hyperpigmented atrophic patch	Multiple hyperpigmented atrophic patches	Diffuse hyperpigmented atrophic patches	Solitary violaceous atrophic patch
Symptoms (itching)		+		+		+
Possible triggers						
Microtrauma (daily shaving)	+	+	+	+		+
Mustard oil massage				+	+	+
Dermoscopy						
Brown to violaceous background	+	+	+	+	+	+
Significantly reduced hair follicles	+	+	+	+	+	+
Cicatricial alopecia	+	+	+	+	+	+
Perifollicular scaling	+	+	+		+	+
Faint background/blotchy erythema	+	+		+	+	+
Brown dots	+		+	+	+	
Whitish scar-like areas		+	+	+		
Prominent pigment clumps	+					+
Peripilar cast		+				+
Pigment pseudo-network	+		+			
Treatment						
Hydroxychloroquine	+	+	+	+		+
Intralesional triamcinolone acetonide	+	+				
Oral steroids	+			+	+	+
Topical steroids	+		+			
Topical calcineurin inhibitor	+	+	+	+	+	+

## Discussion

The exact etiopathogenesis is unknown. An autoimmune reaction against the follicular antigens mediated by T-cells may be the underlying mechanism [5]. Its occurrence following trauma or a surgical procedure further favors this mechanism [6]. None of our patients had any such history; however, all the men described regular shaving. The young girl and two men described regular mustard oil massages over the face that might have irritated and damaged the facial skin [7]. There is a possibility of continuous microtrauma due to shaving and mustard oil application acting as triggers, leading to an abnormal T-cell response against the hair follicle antigens and the development of facial LPP in all our patients.

The clinical differential diagnosis includes linear lichen-planus, localized morphea, benign lichenoid keratosis, lichen striatus, lichen planus pigmentosus, and lupus profundus. There was no history of photosensitivity and direct immunofluorescence (DIF) was negative in all the patients. The age group, lack of erythematous-to-dull red scaly papules, and presence of a localized perifollicular inflammatory infiltrate on histopathology ruled out lichen striatus and lichen planus [8]. The morphology of the lesions and a lack of history of a pre-existing skin lesion like lentigo, actinic keratosis, and seborrheic keratosis ruled out benign lichenoid keratosis [9]. Fixed drug reaction and local steroid-induced atrophy were ruled out, as there was no prior oral, topical, or intralesional drug treatment history. The presence of atrophy clinically, a lack of follicular openings and hem-like pigment, and a folliculocentric involvement in histopathology (HPE) ruled out lichen planus pigmentosus [10]. Linear morphea was also ruled out histopathologically.

There are no treatment guidelines for LPP. All our patients received hydroxychloroquine, potent topical and intralesional steroids, and/or oral steroids. Patient 3 was lost to follow-up and others reported a favorable, albeit suboptimal, response in the form of no progression of the lesions after treatment initiation. No new lesion or hair regrowth was observed in any of the patients at the nine-month follow-up, signifying the importance of halting the disease process in a timely manner, as the atrophy/hair loss may be permanent. Once the disease is dormant and inactive, hair transplantation may be reserved as a last resort for males, and hyaluronic acid fillers may also be used for a better cosmetic outcome.

## Conclusions

This case series highlights the dermoscopy and clinico-histopathological profile of a rare clinical presentation of LPP. Its diagnosis requires a high degree of suspicion in a patient presenting with hyperpigmented atrophic macules on the face. The treatment is difficult and aims to halt the disease progression and/or hair regrowth, which is seldom achieved, and prevent significant morbidity and anxiety in the patient.
